# Steroids from the Soft Coral *Sinularia crassa*

**DOI:** 10.3390/md10020439

**Published:** 2012-02-16

**Authors:** Chih-Hua Chao, Kuei-Ju Chou, Chiung-Yao Huang, Zhi-Hong Wen, Chi-Hsin Hsu, Yang-Chang Wu, Chang-Feng Dai, Jyh-Horng Sheu

**Affiliations:** 1 Chinese Medicinal Research and Development Center, China Medical University Hospital, Taichung 40402, Taiwan; Email: chaochihhua@hotmail.com; 2 China Medical University, Taichung, 40402, Taiwan; 3 Department of Marine Biotechnology and Resources, National Sun Yat-sen University, Kaohsiung 80424, Taiwan; Email: jzusmile@hotmail.com (K.-J.C.); betty8575@yahoo.com.tw (C.-Y.H.); wzh@mail.nsysu.edu.tw (Z.-H.W.); hsuch@mail.nsysu.edu.tw (C.-H.H.); 4 Asian Pacific Ocean Research Center, National Sun Yat-sen University, Kaohsiung 80424, Taiwan; 5 Graduate Institute of Integrated Medicine, College of Chinese Medicine, China Medical University, Taichung 40402, Taiwan; Email: yachwu@mail.cmu.edu.tw; 6 Institute of Oceanography, National Taiwan University, Taipei, 10617, Taiwan; Email: corallab@ntu.edu.tw

**Keywords:** *Sinularia crassa*, crassarosterosides A–D, crassarosterol A, anti-inflammatory activity, *o*-tolylthiocarbamate

## Abstract

One new sterol, crassarosterol A (**1**), and four new steroidal glycosides, crassarosterosides A–D (**2**–**5**) were isolated from the Formosan soft coral *Sinularia crassa*. The absolute configuration of **1** was determined using the Mosher’s method. The absolute configurations for the sugar moieties of **2**–**5** were determined by HPLC analysis on the *o*-tolylthiocarbamates derived from the liberated sugar after acid hydrolysis. Compounds **2** and **4** could significantly inhibit the expression of pro-inflammatory iNOS protein at 10 µM. In contrast, **1**–**3** were found to stimulate the expression of COX-2 protein at this concentration. Steroids **1** and **4** also showed cytotoxicity toward the selected human liver cancer cells.

## 1. Introduction

**Figure 1:**
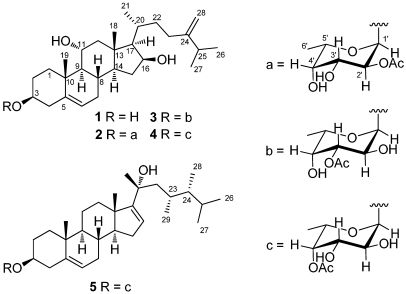
Structures of compounds **1**–**5**.

Soft corals have proven to be a rich source of terpenoids [1]. We previously have isolated a series of bioactive cembrane- [2,3,4] and norcembrane- [5,6,7,8] diterpenoids from the Formosan soft corals of the genus *Sinularia.* Previous chemical investigations on the soft coral *Sinularia crassa* have led to the isolation of structurally unique steroids and cembranoids, from which some have been shown to exhibit anti-inflammatory [9,10] and 5α-reductase inhibitory activities [11], respectively. Our continuing chemical investigation on *S. crassa* has led to the isolation of one new sterol, crassarosterol A (**1**), and four new steroidal glycosides, crassarosterosides A–D (**2**–**5**) (Figure 1). The structures of **1**–**5** have been established by extensive spectroscopic analysis, including 2D NMR (^1^H–^1^H COSY, HMQC, HMBC, and NOESY), chemical methods, and HPLC. The effect of **1**–**4** on the expression of the pro-inflammatory inducible nitric oxide synthase (iNOS) and cyclooxygenase-2 (COX-2) proteins in lipopolysaccharide (LPS)-stimulated RAW264.7 macrophage cells and the cytotoxicity of compounds **1**–**5** against a panel of cancer cell lines including human liver carcinoma (HepG2 and HepG3), human breast carcinoma (MCF-7 and MDA-MB-231), and human lung carcinoma (A-549) were evaluated in order to discover bioactive natural products.

## 2. Results and Discussion

**Figure 2:**
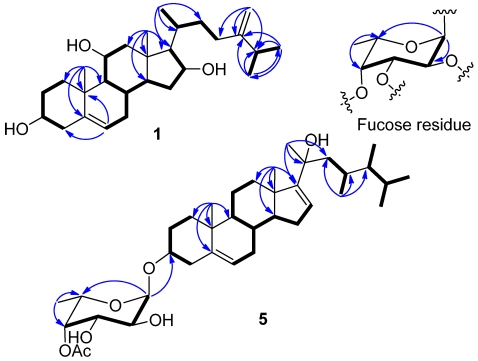
Selected ^1^H−^1^H COSY (▬) and HMBC (→) correlations of **1** and **5** and the fucose residue in **1**–**5**.

**Figure 3:**
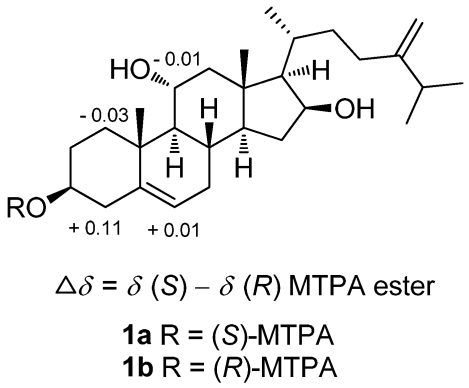
^1^H NMR chemical shift differences of MTPA esters of **1**.

The HRESIMS of crassarosterol A (**1**) exhibited a pseudomolecular ion peak at *m/z* 453.3342 [M + Na]^+^, consistent with a molecular formula of C_28_H_46_O_3_ and requiring six degrees of unsaturation. The ^13^C NMR and DEPT spectroscopic data (Table 1) displayed 28 carbon signals, including five methyls, nine methylenes, ten methines, and four quaternary carbons. The IR spectrum revealed the presence of hydroxy groups (3389 cm^–1^). The carbon resonances at *δ* 141.2 (C), 121.3 (CH), 156.9 (C), and 106.3 (CH_2_) suggested the presence of two double bonds. The above data coupled with the characteristic ^1^H NMR signals for methyl groups at *δ* 0.92 (3H, s), 1.18 (3H, s), 1.04 (3H, d, *J* = 6.8 Hz), and 1.03 (6H, d, *J* = 7.2 Hz) and signals for olefinic protons at *δ*_H_ 5.41 (1H, d, *J* = 5.6 Hz), 4.76 (1H, s), and 4.70 (1H, s) (Table 2) suggested **1** to be a member of the 24-methylenecholesterol class [12,13]. The ^1^H−^1^H COSY correlations allowed the assignment of four separated spin systems (Figure 2). The presence of an sp^2^ methylene substituent at C-24 was further confirmed by the HMBC correlations from H_2_-28 to C-23, C-24, and C-25 (Figure 2). Likewise, the steroidal nucleus was confirmed by the HMBC correlations from H_3_-18 to C-12, C-13, C-14, and C-17 and H_3_-19 to C-1, C-5, C-9, and C-10. The NOE correlations between H_3_-19/H-1β, H-3/H-1α, and H_3_-19/H-11 suggested the α and β orientations for H-3 and H-11, respectively. The absence of an NOE correlation between H_3_-18/H-17 and the presence of the correlation between H-17/H-16, H-16/H-14, and H-14/H-9 suggested the α orientation for H-9, H-14, H-16, and H-17. Moreover, the β orientation for H-20 was evidenced from the NOE correlations between H_3_-18/H-20, H_3_-18/H-12a, and H_3_-21/H-12a. The absolute configuration of **1** was determined by the application of Mosher’s method [14]. Analysis of the ^1^H NMR data of esters **1a** and **1b** resulted in the determination of a 3*S* configuration (Figure 3).

**Table 1:** ^13^C NMR spectroscopic data of compounds **1**−**5**.

position	1*^a^*	2*^b^*	3*^b^*	4*^a^*	5*^a^*
1	39.1, CH_2_	39.2, CH_2_	39.2, CH_2_	39.2, CH_2_	37.1, CH_2_
2	31.8, CH_2_	29.7, CH_2_	29.5, CH_2_	29.7, CH_2_	29.6, CH_2_
3	71.8, CH	78.2, CH	77.8, CH	78.2, CH	78.3, CH
4	42.7, CH_2_	39.1, CH_2_	39.0, CH_2_	39.1, CH_2_	38.8, CH_2_
5	141.2, C	140.6, C	140.7, C	140.5, C	140.3, C
6	121.3, CH	121.7, CH	121.6, CH	121.8, CH	122.1, CH
7	31.9, CH_2_	31.8, CH_2_	31.8, CH_2_	31.8, CH_2_	31.5, CH_2_
8	31.4, CH	31.3, CH	31.3, CH	31.3, CH	30.4, CH
9	56.9, CH	56.8, CH	56.9, CH	56.8, CH	50.3, CH
10	38.1, C	38.3, C	38.3, C	38.3, C	36.9, C
11	68.9, CH	68.9, CH	68.9, CH	68.9, CH	21.0, CH_2_
12	51.4, CH_2_	51.4, CH_2_	51.4, CH_2_	51.4, CH_2_	36.2, CH_2_
13	42.9, C	42.9, C	42.9, C	42.9, C	47.4, C
14	53.7, CH	53.6, CH	53.6, CH	53.6, CH	57.9, CH
15	36.3, CH_2_	36.3, CH_2_	36.3, CH_2_	36.3, CH_2_	31.0, CH_2_
16	72.5, CH	72.5, CH	72.5, CH	72.5, CH	123.8, CH
17	61.2, CH	61.2, CH	61.2, CH	61.2, CH	160.9, CH
18	14.0, CH_3_	14.1, CH_3_	14.1, CH_3_	14.1, CH_3_	18.1, CH_3_
19	19.1, CH_3_	19.0, CH_3_	19.0, CH_3_	19.0, CH_3_	19.3, CH_3_
20	29.6, CH	29.6, CH	29.6, CH	29.6, CH	76.0, C
21	18.2, CH_3_	18.2, CH_3_	18.2, CH_3_	18.2, CH_3_	29.6, CH_3_
22	34.8, CH_2_	34.8, CH_2_	34.7, CH_2_	34.7, CH_2_	49.1, CH_2_
23	31.2, CH_2_	31.2, CH_2_	31.2, CH_2_	31.2, CH_2_	29.6, CH
24	156.9, C	156.9, C	156.9, C	156.9, C	45.5, CH
25	34.1, CH	34.0, CH	34.0, CH	34.0, CH	30.9, CH
26	21.8, CH_3_	21.8, CH_3_	21.8, CH_3_	21.8, CH_3_	20.9, CH_3_
27	21.9, CH_3_	21.9, CH_3_	21.9, CH_3_	21.9, CH_3_	21.5, CH_3_
28	106.3, CH_2_	106.3, CH_2_	106.3, CH_2_	106.4, CH_2_	11.6, CH_3_
29					15.7, CH_3_
1’		97.4, CH	94.7, CH	97.2, CH	97.2, CH
2’		74.0, CH	68.6, CH	70.1, CH	70.1, CH
3’		66.9, CH	72.1, CH	69.5, CH	69.5, CH
4’		70.9, CH	72.4, CH	73.0, CH	73.0, CH
5’		65.8, CH	65.3, CH	65.2, CH	65.2, CH
6’		16.0, CH_3_	16.1, CH_3_	16.2, CH_3_	16.2, CH_3_
OAc		170.9, C	171.5, C	171.3, C	171.3, C
		21.2, CH_3_	21.1, CH_3_	20.8, CH_3_	20.8, CH_3_

*^a^* Spectra were measured in CDCl_3_ (100 MHz); *^b^* Spectra were measured in CDCl_3_ (125 MHz).

Analysis of the HRESIMS and ^13^C NMR spectroscopic data of crassarosteroside A (**2**) suggested a molecular formula of C_36_H_58_O_8_. The IR spectrum of **2** showed the presence of hydroxy (3461 cm^−^^1^) and carbonyl (1741 cm^−^^1^) groups. The latter was identified as an acetoxy group according to the carbon resonances at *δ* 170.9 (C) and 21.2 (CH_3_) (Table 1). The ^1^H NMR spectroscopic data of **2** showed characteristic methyl signals at *δ* 0.92 (3H, s), 1.18 (3H, s), 1.04 (3H, d, *J* = 6.8 Hz), 1.03 (6H, d, *J* = 7.0 Hz), 5.41 (1H, d, *J* = 5.6 Hz), 4.76 (1H, s), and 4.70 (1H, s) (Table 2), revealing that **2** has the same 24-methylenecholesterol skeleton as that of **1**. By excluding the steroidal moiety and the acetoxy group, the remaining six carbons [δ 97.4 (CH), 74.0 (CH), 66.9 (CH), 70.9 (CH), 65.8 (CH), and 16.0 (CH_3_)] were ascribed to the presence of a 6’-deoxyhexose residue. The sugar residue was deduced as an α-fucopyranose on the basis of 2D NMR analysis (Figure 2) and the coupling constants of ^3^*J*_H-1’,H-2’_ (4.0 Hz), ^3^*J*_H-2’,H-3’_ (10.0 Hz), ^3^*J*_H-3’,H-4’_ (3.5 Hz), and ^3^*J*_H-4’,H-5’_ (<1 Hz) (Table 2) [12,15,16]. The acetoxy group attached at C-2’ of the α-fucose residue was evidenced from the downfield chemical shift of H-2’ (*δ* 5.07), which was confirmed by the HMBC correlations from this proton to the acetate carbonyl carbon. The HMBC correlation from H-1’ to C-3 revealed that the α-fucose residue was attached at C-3 of the steroidal aglycone. The absolute configuration of the sugar moiety in **2** was determined by reversed phase HPLC analysis of its *o*-tolylthiocarbamate [17]. The liberated fucose from acid hydrolysis of **2** was treated with L-cysteine methyl ester followed by reaction with *o*-tolylisothiocyanate to afford the corresponding *o*-tolylthiocarbamate derivative. The retention time of the liberated sugar derivative by HPLC analysis was found to be consistent with that of a standard L-fucose derivative. 

Crassarosteroside B (**3**) gave the same molecular formula as that of **2** based on the analysis of the HRESIMS and ^13^C NMR spectroscopic data (Table 1). The NMR spectroscopic data of **3** were similar to those of **2**, with some exceptions for those of the sugar residue. An HMBC correlation from the anomeric proton at *δ* 5.12 (H-1’) to the carbon signal at *δ* 77.8 (C-3) connected the fucose residue to C-3 of the steroidal aglycone. The downfield proton chemical shift at *δ* 4.87 (1H, dd, *J* = 10.5, 4.0 Hz) was ascribed to the presence of an acetoxy group at C-3’ (Table 2). The detailed 2D NMR analysis confirmed the above elucidation. Likewise, the L-fucose residue was deduced according to RP HPLC analysis of the corresponding *o*-tolylthiocarbamate as described above.

Crassarosteroside C (**4**) was assigned the same molecular formula as those of **2** and **3**. A comparison of NMR spectroscopic data of **4** with those of **2** and **3** revealed that an acetoxy group should be located at C-4’ of the fucose residue (Table 1 and Table 2). This was evidenced by the ^1^H NMR shift of H-4’ at 5.20 (1H, d, *J* = 2.8 Hz). In the same manner, RP HPLC anslysis of the corresponding *o*-tolylthiocarbamate derived from the hydrolyte of **4** allowed the determination of the L-fucose moiety. 

The HRESIMS and ^13^C NMR spectroscopic data of crassarosteroside D (**5**) established a molecular formula of C_37_H_60_O_7_. The presence of an acetoxy group was evidenced by the ^1^H NMR signal at δ 2.17 (3H, s) (Table 1) and ^13^C NMR signals at δ 171.3 (C) and 20.8 (CH_3_) (Table 2) as well as the IR absorption band at 1737 cm^−1^. The NMR spectroscopic data for the sugar moiety of **5** were quite similar to those of **4**, suggesting that they shared the same 4’-*O*-acetylfucose residue. Except for the sugar moiety, the remaining 29 carbon signals as well as the characteristic methyl signals at *δ* 1.00 (3H, s), 1.05 (3H, s), 1.38 (3H, s), 0.86 (3H, d, *J* = 6.4 Hz), 0.89 (3H, d, *J* = 6.4 Hz), 0.76 (3H, d, *J* = 6.8 Hz), and 0.78 (3H, d, *J* = 6.4 Hz) (Table 2) revealed that the aglycone of **5** should have a C_29_ steroidal skeleton [18]. The 23,24-dimethyl-20-hydroxy side chain was deduced by the ^1^H−^1^H COSY correlations from H_2_-22 to H_3_-29 through H-23, from H-24 to both H_3_-26 and H_3_-27, and from H-24 to H_3_-28 as well as the HMBC correlations from H_3_-21 to C-17, C-20, and C-22 and H_3_-29 to C-22, C-23, and C-24. This rare steroidal side chain is the same as that of sarcophytosterol isolated previously by us from the soft coral *Lobophytum sarcophytoides* [18]. The NMR spectroscopic data for the aglycone moiety of **5** are almost the same as those of sarcophytosterol, except for some minor variations in ^1^H and ^13^C chemical shifts at C-2, C-3, and C-4 between both compounds. This is due to the attachment of the sugar residue at C-3 of the steroidal aglycone. Similarly, HPLC analysis of the relevant *o*-tolylthiocarbamate derived from the hydrolysis of **5** suggested the presence of L-fucose.

**Table 2:** ^1^H NMR spectroscopic data of compounds **1**−**5**.

＃	1, *δ*_H_ (*J* in Hz)*^a^*	2, *δ*_H_ (*J* in Hz)*^b^*	3, *δ*_H_ (*J* in Hz)*^b^*	4, *δ*_H_ (*J* in Hz)*^a^*	5, *δ*_H_ (*J* in Hz)*^a^*
1	a: 2.55, dt	a: 2.58, dt	a: 2.56, dt	a: 2.58, dt	a: 1.86, m
	(13.6, 3.6)	(13.5, 3.5)	(13.5, 3.5)	(14.0, 3.6)	
	b: 1.16, m	b: 1.16, m	b: 1.16, m	b: 1.16, m	b: 1.10, m
2	a: 1.81, m	a: 1.85, m	a: 1.79, m	a: 1.81, m	a: 1.89, m
	b: 1.58, m	b: 1.65, m	b: 1.66, m	b: 1.64, m	b: 1.60, m
3	3.53, m	3.51, m	3.43, m	3.49, m	3.49, m
4	a: 2.30, m	a: 2.36, m	2.24, m	a: 2.36, m	a: 2.36, m
	b: 2.26, m	b: 2.26, m		b: 2.26, m	b: 2.24, m
6	5.41, d (5.6)	5.41, d (5.5)	5.40, d (5.5)	5.41, d (5.6)	5.38, br d (3.2)
7	a: 1.99, m	a: 1.99, m	a: 1.98, m	a: 1.99, m	a: 2.01, m
	b: 1.54, m	b: 1.56, m	b: 1.54, m	b: 1.54, m	b: 1.61, m
8	1.50, m	1.50, m	1.49, m	1.50, m	1.66, m
9	0.99, m	0.99, m	0.97, m	0.98, m	1.01, m
11	4.07, td	4.07, m	4.06, m	4.07, td	1.59, m
	(10.8, 4.8)			(10.8, 4.4)	
12	a: 2.31, m	a: 2.31, m	a: 2.31, m	a: 2.31, m	a: 2.10, m
	b: 1.18, m	b: 1.18, m	b: 1.18, m	b: 1.18, m	b: 1.59, m
14	0.98, m	0.98, m	0.98, m	0.98, m	1.41, m
15	a: 2.24, m	a: 2.24, m	a: 2.23, m	a: 2.23, m	a: 2.08, m
	b: 1.17, m	b: 1.16, m	b: 1.17, m	b: 1.16, m	b: 1.87, m
16	4.40, m	4.40, m	4.40, m	4.40, m	5.50, br s
17	1.07, m	1.07, m	1.07, m	1.07, m	
18	0.92, s	0.92, s	0.92, s	0.92, s	1.00, s
19	1.18, s	1.18, s	1.17, s	1.18, s	1.05, s
20	1.86, m	1.86, m	1.86, m	1.87, m	
21	1.04, d (6.8)	1.04, d (6.8)	1.04, d (6.5)	1.04, d (6.4)	1.38, s
22	a: 1.68, m	a: 1.68, m	a: 1.68, m	a: 1.67, m	a: 1.59, m
	b: 1.22, m	b: 1.22, m	b: 1.22, m	b: 1.22, m	b: 1.48, m
23	a: 2.18, m	a: 2.18, m	a: 2.18, m	a: 2.18, m	1.82, m
	b: 1.95, m	b: 1.95, m	b: 1.95, m	b: 1.95, m	
24					1.06, m
25	2.24, m	2.25, m	2.25, m	2.25, m	1.42, m
26	1.03, d (7.2)	1.03, d (7.0)	1.03, d (7.0)	1.03, d (6.8)	0.86, d (6.4)
27	1.03, d (7.2)	1.03, d (7.0)	1.03, d (7.0)	1.03, d (6.8)	0.89, d (6.4)
28	a: 4.76, s	a: 4.76, s	a: 4.76, s	a: 4.76, s	0.76, d (6.8)
	b: 4.70, s	b: 4.70, s	b: 4.70, s	b: 4.70, s	
29					0.78, d (7.2)
1’		5.02, d (4.0)	5.12, d (4.0)	5.04, d (4.0)	5.04, d (4.0)
2’		5.07, dd (10.0, 4.0)	4.02, m	3.93, dd (10.0, 4.0)	3.93, dd (10.0, 4.0)
3’		3.91, ddd (11.5,10.0, 3.5)	4.87, dd (10.5, 4.0)	3.74, br d (10.0)	3.74, dd (10.0, 2.4)
4’		3.84, br s	3.82, br s	5.20, d (2.8)	5.21, d (2.4)
5’		4.11, q (7.0)	4.12, q (6.5)	4.12, q (6.4)	4.12, q (6.4)
6’		1.25, d (7.0)	1.27, d (6.5)	1.13, d (6.4)	1.14, d (6.4)
OAc		2.18, s	2.15, s	2.17, s	2.17, s
3’-OH		1.90, d (11.5)			
4’-OH		1.89, br s	2.18, br s		

*^a^* Spectra were measured in CDCl_3_ (400 MHz); *^b^* Spectra were measured in CDCl_3_ (500 MHz).

**Figure 4:**
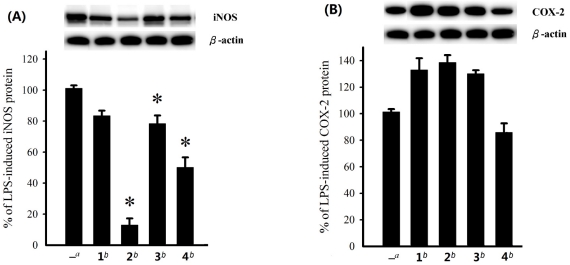
Effect of compounds **1**–**4** at 10 μM on the LPS-induced pro-inflammatory iNOS and on COX-2 protein expression of RAW264.7 macrophage cells by immunoblot analysis. (**A**) Immunoblots for iNOS and *β*-actin, and relative density of iNOS. (**B**) Immunoblots for COX-2 and *β*-actin, and relative density of COX-2. The values are means ± SEM (*n* = 6). The relative intensity of the LPS alone stimulated group was taken as 100%. Under the same experimental conditions, 10 μM CAPE (caffeic acid phenethyl ester; Sigma Chemical Company, St. Louis, MO) reduced the levels of the iNOS and COX-2 protein to 0.8 ± 4.5% and 75.6 ± 12.2%, respectively, relative to the control cells stimulated with LPS. *Significantly different from LPS alone stimulated group (*P < 0.05).

The absolute configuration of sterol **1** has been established by Mosher’s method in the present work. On the basis of biogenesis, the steroidal moieties of the glycosides **2**–**5** should possess the same absolute configurations as shown in the formulae. Cytotoxicity of steroids **1**–**5** against HepG2, HepG3, MCF-7, MDA-MB-231, and A-549 cancer cell lines was evaluated. The results showed that **1** exhibited cytotoxicity toward HepG2 cancer cell line with an IC_50_ value of 14.9 µM, while **4** also showed cytotoxicity toward HepG2 and HepG3 cell lines with IC_50_ values of 17.6 and 18.9 µM, respectively. The other compounds were found to be inactive (IC_50_ > 20 μM) toward the above cancer cell lines after 72 h exposure. The effect of steroids **1**–**4** on the expression of pro-inflammatory iNOS and COX-2 proteins in RAW264.7 macrophage cells stimulated with LPS was also evaluated using immunoblot analysis. At a concentration of 10 µM (Figure 4), steroid **2** was found to significantly reduce the level of iNOS protein to 12.9 ± 4.3% and **4** could reduce the iNOS espression to 50.1 ± 6.3%. However, these compounds could not effectively reduce the level of COX-2. On the contrary, **1**–**3** were found to stimulate the expression of COX-2.

## 3. Experimental Section

### 3.1. General Experimental Procedures

The melting point was determined using a Fisher-Johns melting point apparatus. Optical rotations were determined with a JASCO P1020 digital polarimeter. IR spectrum was obtained on a JASCO FT/IR-4100 spectrophotometer. The NMR spectra were recorded on a Varian 400 MR NMR or Varian Unity INOVA 500 FT-NMR instrument at 400 or 500 MHz for ^1^H (referenced to TMS, *δ*_H_ 0.00 ppm for CDCl_3_) and 100 or 125 MHz for ^13^C (referenced to *δ*_C_ 77.0 for CDCl_3_). ESIMS and HRESIMS were recorded by ESI FT-MS on a Bruker APEX II mass spectrometer. Silica gel 60 (Merck, 230−400 mesh) and LiChroprep RP-18 (Merck, 40–63 μm) were used for column chromatography. Precoated silica gel plates (Merck, Kieselgel 60 F_254_, 0.25 mm) and precoated RP-18 F_254S_ plates (Merck, 1.05560) were used for TLC analyses. High-performance liquid chromatography (HPLC) was performed on a Hitachi L-7100 pump equipped with a Hitachi L-7400 UV detector at 210 nm and a semi-preparative reversed-phase column (Merck, Hibar Purospher RP-18e, 5 μm, 250 × 10 mm).

### 3.2. Animal Material

The soft coral *Sinularia* *crassa* was collected by hand using scuba off the coast of Sansiantai, Taitung county, Taiwan, in July 2008, at a depth of 10 m, and was stored in a freezer. This soft coral was identified by one of the authors (C.-F.D.). A voucher specimen (specimen no. SST-03) was deposited in the Department of Marine Biotechnology and Resources, National Sun Yat-sen University. 

### 3.3. Extraction and Isolation

The frozen bodies of *S. crassa* (1.1 kg fresh wt) were minced and extracted with EtOH (3 × 2 L). The organic extract was concentrated to an aqueous suspension and was further partitioned between EtOAc and H_2_O. The EtOAc extract (17.0 g) was fractionated by open column chromatography on silica gel using *n*-hexane–EtOAc and EtOAc–MeOH mixtures of increasing polarity to yield 32 fractions. Fractions 25, eluting with EtOAc–MeOH (8:1), was further separated by silica gel column chromatography with gradient elution (*n*-hexane–acetone, 8:1 to 2:1) to yield four subfractions (25A–25D). Subfraction 25B was subjected to RP-18 column chromatography (MeOH–H_2_O, gradient, 50–90%), and subsequently purified by RP-18 HPLC (CH_3_CN–H_2_O, 65%) to obtain compounds **1** (6.6 mg) and **5** (1.2 mg). Compound **4** (1.8 mg) was obtained from subfraction 25C using RP-18 HPLC (CH_3_CN–H_2_O, 65%). In the same manner, Subfraction 25D was fractionated over RP-18 gel using gradient elution (MeOH–H_2_O, gradient, 50–90%) to yield two subfractions (25D-1 and 25D-2). Subfraction 25D-2 was separated by RP-18 HPLC (CH_3_CN–H_2_O, 85%) to yield compounds **2** (1.8 mg) and **3** (1.6 mg).

Crassarosterol A (**1**): white powder; [α]^24^_D_ −45 (*c* 0.66, CHCl_3_); IR (KBr) *v*_max_ 3389, 2962, 2925, 2854, 1462, 1048, 1024 cm^−1^; ^13^C NMR and ^1^H NMR data, see Table 1 and Table 2; ESIMS *m/z* 453 [M + Na]^+^; HRESIMS *m/z* 453.3342 [M + Na]^+^ (calcd for C_28_H_46_O_3_Na, 453.3344).

Crassarosteroside A (**2**): white powder; [α]^24^_D_−34 (*c* 0.18, CHCl_3_); IR (KBr) *v*_max_ 3461, 2960, 2928, 2868, 1741, 1467, 1377, 1244, 1077, 1030 cm^−^^1^; ^13^C NMR and ^1^H NMR data, see Table 1 and Table 2; ESIMS *m/z* 641 [M + Na]^+^; HRESIMS *m/z* 641.4027 [M + Na]^+^ (calcd for C_36_H_58_O_8_Na, 641.4029).

Crassarosteroside B (**3**): white powder; [α]^24^_D_−17 (*c* 0.16, CHCl_3_); IR (KBr) *v*_max_ 3388, 2963, 2930, 2857, 1732, 1458, 1375, 1258, 1041 cm^−^^1^; ^13^C NMR and ^1^H NMR data, see Table 1 and Table 2; ESIMS *m/z* 641 [M + Na]^+^; HRESIMS *m/z* 641.4026 [M + Na]^+^ (calcd for C_36_H_58_O_8_Na, 641.4029).

Crassarosteroside C (**4**): white powder; [α]^24^_D_−52 (*c* 0.18, CHCl_3_); IR (KBr) *v*_max_ 3426, 2960, 2930, 2859, 1735, 1461, 1375, 1247, 1074, 1033 cm^−^^1^; ^13^C NMR and ^1^H NMR data, see Table 1 and Table 2; ESIMS *m/z* 641 [M + Na]^+^; HRESIMS *m/z* 641.4026 [M + Na]^+^ (calcd for C_36_H_58_O_8_Na, 641.4029).

Crassarosteroside D (**5**): white powder; [α]^24^_D_−45 (*c* 0.12, CHCl_3_); IR (KBr) *v*_max_ 3440, 2960, 2925, 2855, 1737, 1461, 1377, 1244, 1074, 1036 cm^−^^1^; ^13^C NMR and ^1^H NMR data, see Table 1 and Table 2; ESIMS *m/z* 639 [M + Na]^+^; HRESIMS *m/z* 639.4234 [M + Na]^+^ (calcd for C_37_H_60_O_7_Na, 639.4237).

### 3.4. Preparation of (***S***)-and (***R***)-MTPA Esters of ***1***

To a solution of **1** (1.0 mg) in pyridine (0.4 mL) was added (*R*)**-**MTPA chloride (25 μL), and the mixture was allowed to stand for 3 h at room temperature. The reaction was quenched by the addition of 1.0 mL of H_2_O, and the mixture was subsequently extracted with EtOAc (3 × 1.0 mL). The EtOAc layers were combined, dried over anhydrous MgSO_4_, and evaporated. The residue was subjected to short silica gel column chromatography using *n*-hexane−EtOAc (3:1) to yield the (*S*)-MTPA ester, **1a** (0.7 mg). The same procedure was used to prepare the (*R*)-MTPA ester, **1b** (1.0 mg from 1.0 mg of **1**), with (*S*)-MTPA chloride. Selected ^1^H NMR (CDCl_3_, 400 MHz) of **1a**: δ 7.41−7.52 (5H, m, Ph), 5.48 (1H, br d, *J* = 6.0 Hz, H-6), 4.89 (1H, m, H-3), 4.76 (1H, s, H-28a), 4.70 (1H, s, H-28b), 4.41 (1H, m, H-16), 4.05 (1H, m, H-11), 3.57 (3H, s, O*Me*), 2.62 (1H, br d, *J* = 14.0 Hz, H-1a), 2.48 (1H, m, H-4a), 1.85 (1H, m, H-2a), 1.17 (3H, s, H_3_-19), 1.03 (6H, d, *J* = 7.2 Hz, H_3_-26 and 27), 0.92 (3H, s, H_3_-18); selected ^1^H NMR (CDCl_3_, 400 MHz) of **1b**: δ 7.41−7.53 (5H, m, Ph), 5.47 (1H, br d, *J* = 5.2 Hz, H-6), 4.89 (1H, m, H-3), 4.76 (1H, s, H-28a), 4.70 (1H, s, H-28b), 4.41 (1H, m, H-16), 4.06 (1H, m, H-11), 3.57 (3H, s, O*Me*), 2.65 (1H, br d, *J* = 13.6 Hz, H-1a), 2.37 (1H, m, H-4a), 1.77 (1H, m, H-2a), 1.17 (3H, s, H_3_-19), 1.03 (6H, d, *J* = 7.2 Hz, H_3_-26 and 27), 0.92 (3H, s, H_3_-18).

### 3.5. Determination of Sugar Configuration

Authentic samples of D-fucose and L-cysteine methyl ester hydrochloride (each 0.5 mg) were dissolved in pyridine (0.1 mL) and heated at 60 °C for 1 h. To this mixture was added *o*-tolylisothiocyanate (0.5 mg in 0.1 mL pyridine) and heated at 60 °C for additional 1 h. The reaction mixture was directly analyzed by reversed-phase HPLC (Mightysil RP-18 GP column; 4.6 × 250 nm; 25% CH_3_CN in 50 mM H_3_PO_4_; 0.8 mL/min; 35 °C) and detected at 250 nm to give the retention time of the *o*-tolylthiocarbamate of sugar. The retention of the *o*-tolylthiocarbamate derived from L-fucose, L-cysteine methyl ester, and *o*-tolylisothiocyanate was obtained by the same manner.

A solution of the glycoside (0.4 mg for each) in 0.6 M HCl/dioxane (1:1 v/v, 0.2 mL) was heated at 80 °C for 24 h. After cooling, the solution was neutralized with Amberlite IRA-400, and the resin was removed by filtration. The filtrate was extracted with EtOAc. The aqueous layer was dried *in vacuo* and the afforded residue was dissolved in pyridine (0.1 mL) containing L-cysteine methyl ester (0.5 mg), followed by heating at 60 °C for 1 h. A 0.1 mL solution of *o*-tolylisothiocyanate (0.5 mg) in pyridine was added to the mixture, which was heated at 60 °C for additional 1 h, to yield the corresponding *o*-tolylthiocarbamate derivative. Reverse phase HPLC analysis of the *o*-tolylthiocarbamate derivatives derived from the hydrolytes of the glycosides **2**–**5** showed peaks at 28.2, 28.0, 28.1, and 27.9 min, respectively, while the *t*_R_ values for standard L-fucose and D-fucose derivatives were observed at 28.0 and 25.4 min, respectively, suggesting the presence of a L-fucose residue in **2**–**5**.

### 3.6. Cytotoxicity Testing

Cell lines were purchased from the American Type Culture Collection (ATCC). Compounds were assayed for cytotoxicity against human liver carcinoma (HepG2 and HepG3), human breast carcinoma (MCF-7 and MDA-MB-231), and human lung carcinoma (A-549) cells using the 3-(4,5-dimethylthiazol-2-yl)-2,5-diphenyltetrazolium bromide (MTT) method [19]. Freshly trypsinized cell suspensions were seeded in 96-well microtiter plates at densities of 5000–10000 cells per well with tested compounds added from DMSO-diluted stock. After 3 days in culture, attached cells were incubated with MTT (0.5 mg/mL, 1 h) and subsequently dissolved in DMSO. The absorbency at 550 nm was then measured using a microplate reader. The IC_50_ is the concentration of agent that reduced cell growth by 50% under the experimental conditions. 

### 3.7. *In Vitro* Anti-Inflammatory Assay

Macrophage (RAW264.7) cell line was purchased from ATCC. In vitro anti-inflammatory activities of tested compounds were measured by examining the inhibition of lipopolysaccharide (LPS) induced upregulation of iNOS and COX-2 proteins in macrophage cells using western blotting analysis [20,21].

## 4. Conclusions

Prior investigation of the genus *S* *inularia* reported on several steroidal glycosides, however, all of these were found to possess a 24-methylene substituted side chain [22,23]. **5** is the first example of steroidal glycoside with a 23,24-dimethyl substitited side chain from soft coral of the genus *S* *inularia*. Our biological data reveal that 2′-*O*-acetyl-L-fucose functionality plays an important role toward the inhibition of the pro-inflammatory iNOS. Steroidal glycoside **2** could be a useful anti-inflammatory agent, while steroids **1** and **4** have shown inhibitory activity toward the selected human liver cancer cells.
